# Hepatic Hydatid Cyst With Cystobiliary Communication and Cystoduodenal Fistula

**DOI:** 10.7759/cureus.17473

**Published:** 2021-08-26

**Authors:** Oseen Shaikh, Naveen Kumar Gaur, Chellappa Vijayakumar, Uday Kumbhar, Raja Kalayarasan

**Affiliations:** 1 Surgery, Jawaharlal Institute of Postgraduate Medical Education and Research, Puducherry, IND; 2 Surgical Gastroenterology, Jawaharlal Institute of Postgraduate Medical Education and Research, Puducherry, IND

**Keywords:** hepatic hydatid cyst, echinococcus, cystoduodenal fistula, propofol, cystobiliary communication

## Abstract

The liver is the most common site affected by hydatid disease. Hepatic hydatid cyst (HHC) with cystoduodenal fistula is an unusual and infrequent complication. We present a 48-year-male diagnosed with an HHC with cystobiliary communication (CBC) and cystoduodenal fistula. The patient underwent partial cystectomy. Intraoperative demonstration of CBC was done with injection propofol, followed by primary closure of the CBC. The duodenal fistula was closed primarily with an omental patch, also known as a Graham patch. The patient improved well without any complications, and there was no recurrence of the symptoms for the subsequent five-month follow-up.

## Introduction

Hepatic hydatid cyst (HHC) is a common disease caused by Echinococcus. HHC can be of varying size and usually asymptomatic. HHC can get complicated with the formation of cystobiliary communication (CBC), rupture into the peritoneal cavity, and bronchi [1,2]. Fistulization of the cyst into the duodenum is an unusual complication [3]. Imaging usually helps in the diagnosis of the disease due to its classical radiological features. Sometimes there may not be any classical imaging features of the cyst if the cyst is infected. Serology is usually positive in HHC and is helpful for diagnosis. Surgical treatment is the gold standard for complicated HHC. Intraoperatively, CBC should be identified meticulously and treated appropriately. We report an unusual case of HHC with CBC and cystoduodenal fistula, which was managed successfully without any complications.

## Case presentation

A 48-year-old male presented in emergency with a history of abdominal mass since childhood. The abdominal mass was gradually progressive in size and rapidly increased in the last two weeks. He also complained of breathlessness for one week associated with abdominal pain. The patient never went to any hospital for the abdominal mass. There was no history of vomiting, altered bowel habits, trauma to the abdomen, melena, and passage of any membranes in the stools. There was no history of cough with sputum. On examination, the patient had tachycardia with normal blood pressure. There was no pallor or jaundice. Abdominal examination showed a large mass of almost 20cm x 20cm occupying the epigastrium, right hypochondrium, left hypochondrium, and umbilical region. It had varying consistency - soft in the upper part and firm in the lower and lateral parts. 

**Figure 1:**
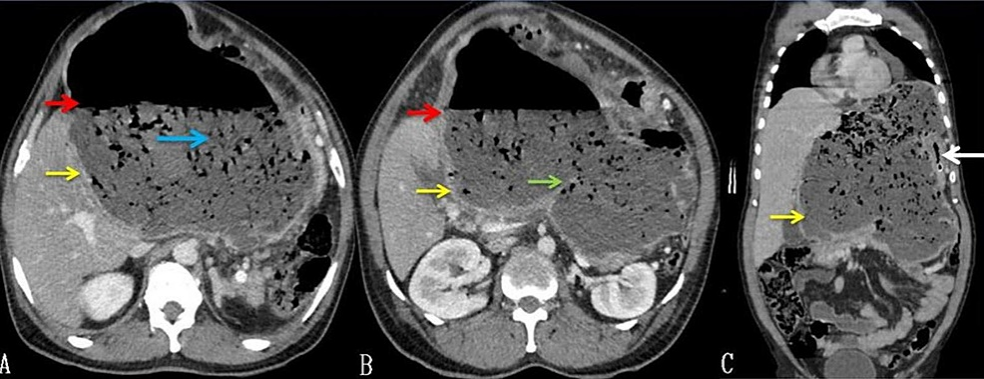
CT of the abdomen (A: axial view, B: axial view, and C: coronal view) A large cyst is seen originating from the left lobe of the liver (yellow arrow) with well defined air-fluid level (red arrow) and debris within the cyst (blue arrow), doubtful septation or membrane (green arrow), stomach being fully compressed (white arrow).

Routine blood investigations, including complete blood counts and renal function tests, were normal. A liver function test revealed mild elevation (174 IU/L) of alkaline phosphatase (ALP). Hydatid serology was positive. Abdominal USG showed the presence of a large hypoechoic cyst with air and debris. Contrast-enhanced CT of the abdomen showed a 20cm x 18cm cyst, arising from the left lobe of the liver having an air-fluid level and few septations or membranes at the inferior aspect of the cyst with debris. The left portal vein was compressed, and the left bile duct was not visualized. The gallbladder had air within it. The common bile duct (CBD) was normal in caliber, and there was no air within it (Figure 1).

**Figure 2:**
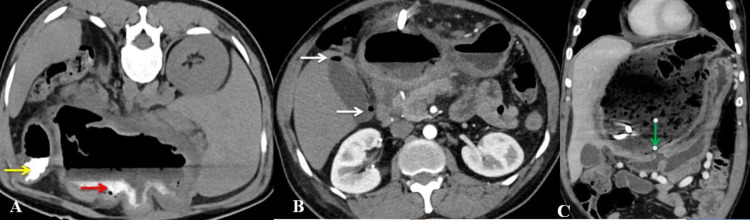
CT of the abdomen A (axial view, prone position): Oral contrast into the stomach (yellow arrow) and the cyst (red arrow) B (axial view): Air within the gallbladder at fundus and neck (white arrows) C (coronal view): Fistulous communication between duodenum and cyst (green arrow)

The cyst's fistulous communication with the duodenum was demonstrated with oral contrast CT scan (Figure 2). The cyst was closely related to the stomach and had displaced the stomach and spleen laterally. The pancreas appeared normal.

As the patient was breathless, a decision was made to put a percutaneous pigtail drain within the cyst to decompress. The percutaneous tube drained about 800 mL of bile-stained foul-smelling fluid with whitish jelly-like thin membranes. The microbiological analysis of the fluid showed the growth of gram-negative organisms, suggestive of an infected cyst. The cystic fluid's carcinoembryonic antigen (CEA) level was 230 ng/mL, and the carbohydrate antigen (CA19-9) was more than 1972 U/mL. However, serum CEA and CA19-9 levels were within the normal range.

**Figure 3:**
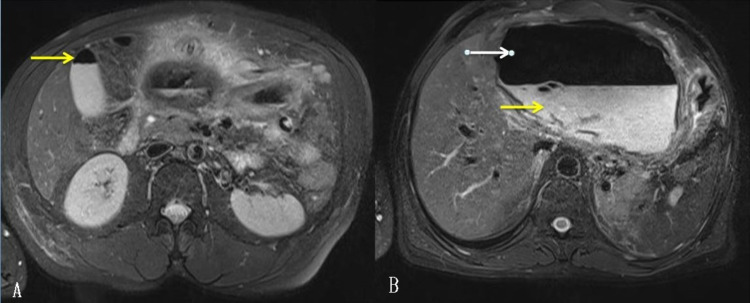
Magnetic resonance cholangiopancreatography (axial view) A: Air in the gallbladder (arrow) B: Hyperintense cystic content (yellow arrow) with air (white arrow) within it.

Magnetic resonance cholangiopancreatography (MRCP) showed a well-defined large cyst of 15cm x 15cm, probably arising from the left lobe of the liver with an air-fluid level in the epigastric region with pigtail inside. CBC could not be demonstrated clearly (Figure 3).

**Figure 4:**
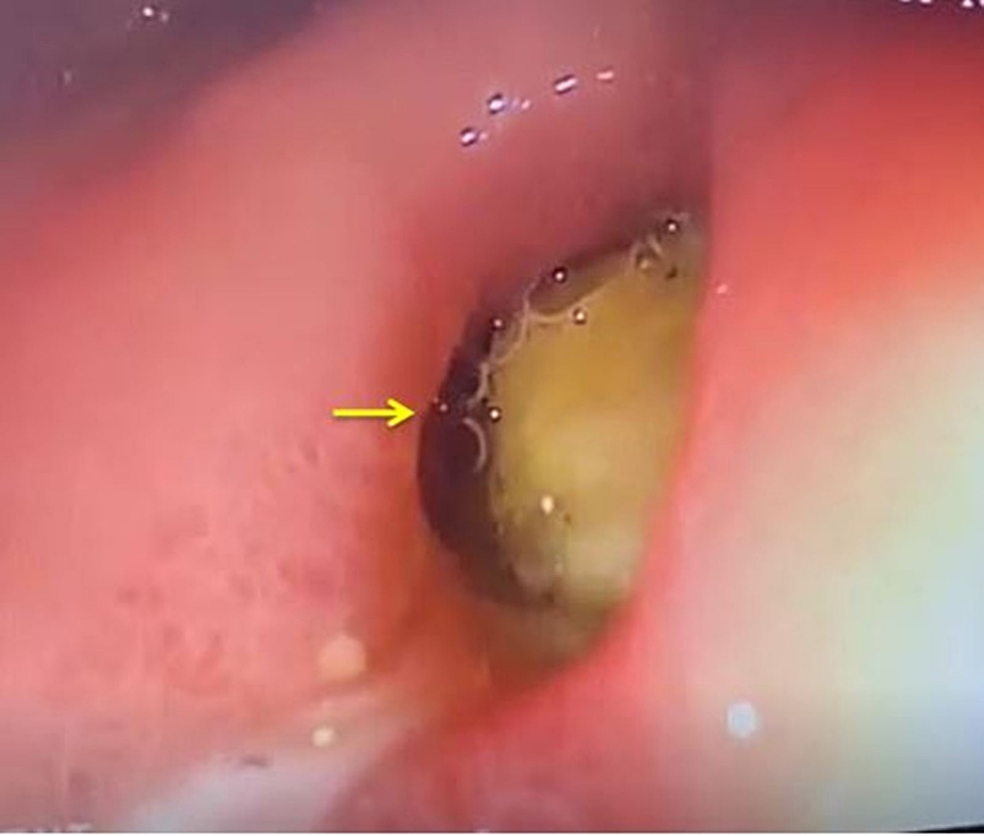
Upper gastrointestinal endoscopy showing fistulous communications of the first part of the duodenum with the cyst (arrow).

Upper gastrointestinal endoscopy showed a bulge along the lesser curvature of the stomach and fistulous opening in the first part of the duodenum (Figure 4).

The patient was prepared for laparotomy and possibly left hepatectomy. After exploration, the abdomen was packed with cetrimide-soaked pads. Intraoperatively, a large cyst was found from the liver's left lobe and had dense adhesions with the stomach, diaphragm, and transverse mesocolon. During the adhesiolysis, there was an inadvertent opening of the cyst wall. Hence the cyst wall was opened for controlled drainage. The inside of the cyst wall was covered with a whitish germinal layer. Hence, the diagnosis of the HHC was confirmed. We proceeded with a partial cystectomy of the HHC. The germinal layer of the residual cyst wall was removed by scraping.

**Figure 5:**
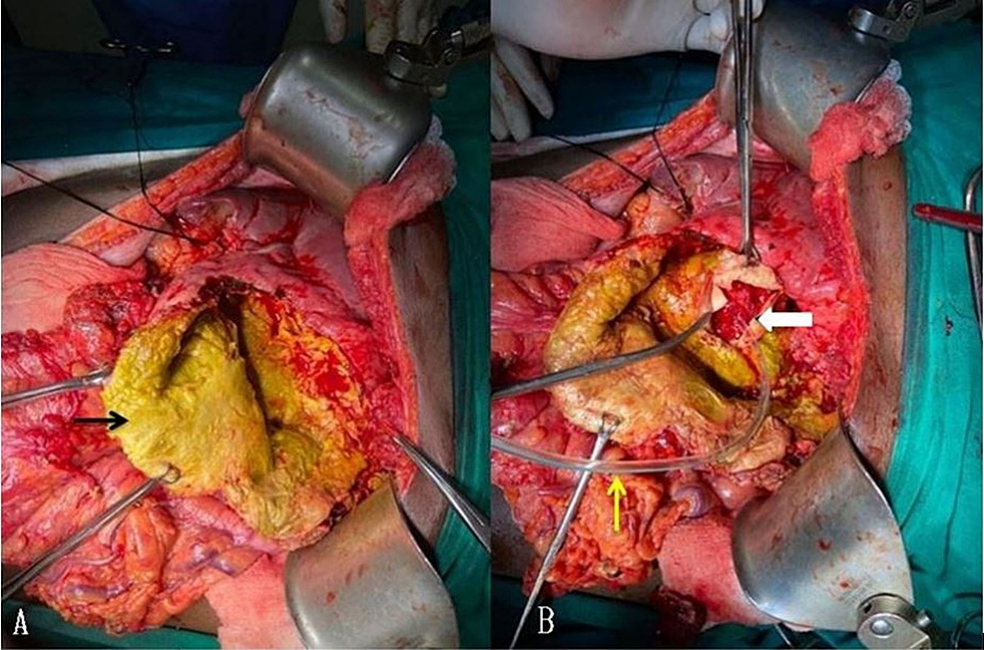
Intraoperative image A: Opened cyst having bile stained wall and germinal layer (black arrow) B: Fistulous communication with the duodenum (white arrow) in which nasogastric tube (yellow arrow) has been passed

A meticulous search for the CBC was done. A cholecystectomy was performed. The cystic duct’s cannulation was done, the catheter advanced into the hepatic duct, and CBD was blocked with a clamp. Propofol was injected through the catheter. Four CBCs were very well demonstrated with leakage of the propofol through the inner surface of the residual cyst wall. All the communications were primarily sutured with polypropylene. A drain was placed into the residual cyst cavity, and omentopexy was done. The cystoduodenal fistula was visualized during the cystectomy (Figure 5).

As the size of the fistula was less than 5 mm, it was closed primarily with an omental patch.

Postoperatively patient improved well without any complications. Orals were started on the fourth postoperative day and escalated gradually. The patient was continued with albendazole for three months. Repeat USG did not show any evidence of the recurrence. Postoperative histopathological features were diagnostic of hydatid cyst.

## Discussion

Hydatid disease is a zoonotic disease caused by *Echinococcus spp, *tapeworms. Although it can affect any organ, the liver is the most common organ [4]. Four species of *Echinococcus* can cause hydatid disease; *Echinococcus granulosus* is the most common. The definitive host for *Echinococcus* is dog, and the intermediate host is sheep. Humans are considered accidental intermediate hosts. Humans get infected from eating raw vegetables infected with dogs’ feces or direct contact with dogs. Few professionals like slaughterhouse workers, shepherds, and butchers are at high risk of hydatid disease.

HHC grows slowly and can be of varying sizes. A mature cyst has the inner germinal layer, a laminated layer, and an adventitial layer. Hydatid fluid is usually clear and colorless. Fluid may be bile stained if CBC is present. HHC is usually asymptomatic. The patient becomes symptomatic once the cyst attains a large size or gets complicated. HHC can get infected or rupture into the biliary tree, or a bronchial tree or peritoneum [1,2]. Rupture into the pericardium or the digestive tract is very rare [3]. Our patient had both CBC and spontaneous cystoduodenal fistula.

CBC occurs when there is a gradual increase in the cyst’s size and a simultaneous increase in intracystic pressure. The intracystic pressure exceeds the ductal pressure, and the cyst wall and the duct weakens at the point of contact. This leads to the formation of CBC, and bile enters into the cyst. These communications are usually small and silent. In our patient, the hydatid fluid, drained by the percutaneous drain, was bile stained, suggestive of CBC, and had a foul smell and suppuration.

Enteric communication of the hydatid cyst is rare. Although HHC commonly ruptures in the stomach and duodenum, it can also involve the esophagus, colon, and small bowel. This leads to incomplete evacuation of the cyst contents and reduction of the cyst pressure. The fistulization can also occur due to the friction of the thick pericyst with the bowel wall. Our patient had spontaneous fistulization to the duodenum. This could have been due to the long duration of the cyst and its erosion into the bowel wall as the cyst had dense adhesion with the stomach and the first part of the duodenum.

If HHC ruptures in the GI tract, patients may pass hydatid membranes in the stool (hydatidorrhea) and hydatid membranes in the vomitus (hydatidemesis). Few patients may have non-specific symptoms [5]. Our patient had only non-specific abdominal pain and progressive abdominal distention, causing breathlessness and discomfort.

Diagnosis of the hydatid cyst is usually by clinical history, imaging, and serological studies. Liver function tests may be normal, or there may be an increase in ALP or gamma-glutamyl transferase in a few patients, which are suggestive of CBC. Many serological tests like immunoelectrophoresis (IEP) and enzyme-linked immunosorbent assay (ELISA) are used for diagnosis and post-treatment surveillance, but all have varying sensitivity and specificity. ELISA test has a sensitivity of 85-98% [6]. There was a mild increase in ALP in our patient, and the ELISA test for serology was positive.

Tumor markers like CA 19-9 and CEA help differentiate cystic lesions of the liver. These tumor markers may be elevated in patients with cystic liver lesions like mucinous cystic neoplasia of the liver (MCN-L) and intraductal pancreatic mucinous neoplasm-biliary type (IPMN-B). However, serum levels of the tumor markers in such cases are usually inconsistent. There are few reports in the literature where CA19-9 was elevated in patients with HHC, suggesting occult CBC. In our patient, serum levels of these tumor markers were normal, but these were highly elevated in the cyst’s fluid. 

Abdominal USG may show anechoic cyst, multiseptate cyst, floating membranes within the cyst, daughter cysts, and calcifications. If the cyst gets secondarily infected, it will lose its characteristic appearance [2]. Based on the USG appearance, these hydatid cysts have been classified by Gharbi et al. into five categories and WHO Informal Working Group on Echinococcosis (WHO-IWGE) into six categories [7,8]. The CT scan of the abdomen shows the location of the cyst, thickness of the wall, presence of daughter cysts, floating membranes, and calcifications if any. Both these imaging techniques usually will not be able to show any CBC if present. The presence of air within the cyst, which both imaging techniques can see, should be highly suggestive of the presence of cystoenteric fistula. In our patient, there was a large hypoechoic cyst with debris and air within it. CT showed debris in the dependent region with an air-fluid level with few membranes in the inferior aspect. The cyst had communication with the duodenum, and there was a presence of air within the gallbladder, with normal CBD without any air. We also hypothesize that the air in the gallbladder was due to duodenal fistulization of the cyst with preexisting CBC. Air must have entered in the biliary radicals and then into the gallbladder through the cystic duct.

MRI is helpful in the diagnosis of HHC. It shows a hypointense pericyst on T1 weighted images, hyperintense cyst on T2 weighted images. The presence of CBC can be suggested by the presence of fat density within the cyst. It provides good visualization of the relationship of the intrahepatic and extrahepatic biliary tree with the cyst [9]. In our case, MRCP showed normal caliber CBD without any apparent communication with the cyst.

Treatment of the HHC involves medical, percutaneous, and surgical methods. Pharmacological treatment of the hydatid cyst includes albendazole and mebendazole, which are given perioperatively. Percutaneous treatment includes percutaneous aspiration, injection and re-aspiration (PAIR), and its modifications. Surgical treatment can be laparoscopic or open techniques, radical or conservative. Radical surgeries include partial pericystectomy, total cystopericystectomy, left hepatectomy, right hepatectomy. Conservative surgery includes cystectomy or also called closed cystectomy. Our patient initially was planned for left hepatectomy due to uncertainty of the diagnosis. During the cyst’s dissection, there was the inadvertent opening of the cyst due to dense adhesions with surrounding organs. Then the cyst was fully decompressed, and the germinal layer’s presence within the cyst confirmed the diagnosis of hydatid cyst. Hence cystectomy was done. 

Intraoperatively, careful inspection of the cyst wall has to be done to see for the presence of CBC. These can be demonstrated by keeping a dry pad on the cyst’s inner surface, applying gentle pressure on the gallbladder, intraoperative cholangiography, methylene blue injection, saline injection, and indocyanine green injection through the cystic duct. As these dyes stain the surface, these can mask the additional leaks [10]. Hence few have used propofol injection intraoperatively into the cystic duct or bile duct to detect a biliary leak [11]. This is based on the White test using a fat emulsion to detect bile leakage, as the emulsion will be easily recognized and easily removed without misleading tissue staining [10]. CBC is usually sutured if they are small. The presence of large communication may need hepaticojejunostomy. We injected propofol through the cystic duct after cholecystectomy, demonstrating CBC clearly, and all the leaks were primarily sutured.

If HHC forms fistula with bowel, the treatment depends will on the site and size of the fistula. There are case reports in the literature where a cyst with colonic fistula was treated by resection, or by creating stoma at the fistula site or by primary closure [12]. If the cyst has communication with the duodenum or stomach, treatment options include primary closure of the fistula with an omental patch, duodenal diverticulization, or pyloric exclusion. We did primary closure of the duodenal fistula with an omental patch.

## Conclusions

HHC can be of varying size, and patients may present later with complications. Infected HHC will lose its imaging characteristics, and it may not be easy to diagnose by imaging. The presence of air in the cyst is highly suggestive of fistulization with the bowel. There may be an elevation in the CA19-9 and CEA within the cystic fluid once CBC is established, creating confusion in the diagnosis with other diseases like MCN-L of liver or IPMN-B. Propofol can be used intraoperatively to check for CBC, as other dyes may stain the surface and mask other leaks. The presence of air in the gallbladder does not necessarily indicate communication of the cyst with the gallbladder. To the best of our knowledge, this is the first case of HHC in the literature having air in the gallbladder, which must have entered through the CBC of the HHC once the duodenal fistulization was established, and the surgeon should be aware of such a possibility.
